# “We’ve all Lost so Much”: The Experiences of Essential Family Caregivers’ Long Term Care Visitations During COVID-19

**DOI:** 10.1093/geroni/igab046.2950

**Published:** 2021-12-17

**Authors:** Charlene Chu, Amanda Yee, Vivian Stamatopoulos

**Affiliations:** 1 University of Toronto, Toronto, Ontario, Canada; 2 University of Toronto, Calgary, Alberta, Canada; 3 University of Ontario Institute of Technology (UOIT), Oshawa, Ontario, Canada

## Abstract

Family caregivers are integral to the care of long-term care (LTC) residents. COVID-19 public health policies initially restricted all essential caregivers from visiting LTC homes. In lieu of in-person visitations, caregivers were allowed technology-based visits then restrictive outdoor visits, followed by indoor visitations. This study aims to illuminate the experiences of essential caregivers’ as they visited their loved ones in LTC during COVID-19’s restrictive policies. We conducted seven caregiver focus groups (N=30) from Ontario and British Columbia, Canada. Thematic analysis and line-by-line coding were completed using NVivo. We found six themes that were common to all the visitation types: 1) “LTC Home disorganization” to facilitate visits and poor communication; 2) “Lack of staffing and resources”; 3) “Mistreatment from staff and management” as caregivers were seen as inconveniences; 4) “Shock and disbelief” when family members first saw their loved ones; 5) “Significant lack of person-centered or family-centered ethos” for example the residents’ needs were ignored such that their cognitive and physical impairments sometimes made visitations impossible, as well as the burden of multiple weekly COVID-19 tests; and, 6) “Collateral damage” in the form of trauma and irreparable harm to the relationships between residents and families. These results emphasized caregivers who ultimately felt betrayed and ignored by the broader healthcare system. Our findings provide an in-depth understanding of how COVID-19 public health policies have impacted the essential caregivers and the long-lasting impacts on residents and caregivers alike. Understanding caregiver’s experiences can inform future pandemic response policies and encourage more person-centered protocols.

